# Protective effect of a sesamin derivative, 3-bis (3-methoxybenzyl) butane-1, 4-diol on ischemic and hypoxic neuronal injury

**DOI:** 10.1186/1423-0127-21-15

**Published:** 2014-02-18

**Authors:** Chien-Wei Hou, Yi-Ling Chen, Shih-Hsien Chuang, Jen-Shu Wang, Kee-Ching Jeng

**Affiliations:** 1 Department of Biotechnology, Yuanpei University, Hsinchu 30015, Taiwan; 2 Department of Small Molecule Drugs, Development Center for Biotechnology, New Taipei 22180, Taiwan; 3 Department of Chinese Medicine, Tzuchi General Hospital, Taichung 42743, Taiwan; 4 Department of Medical Research, Tungs’ Taichung MetroHarbor Hospital, 699 Taiwan Boulevard, Sec.8, Taichung 43503, Taiwan

**Keywords:** Cerebral ischemia, Hypoxia, Neuroprotection, Sesamin derivative, Membrane permeability

## Abstract

**Background:**

Stroke is one of the leading causes of neuronal death. Sesamin is known for neuroprotection by its antioxidant and anti-inflammatory properties but it lacks blood–brain barrier (BBB) activity. A panel of sesamin derivatives was screened and 3-bis (3-methoxybenzyl) butane-1,4-diol (BBD) was selected for high BBB activity and tested for its neuroprotective effect.

**Methods:**

The focal cerebral ischemia of Sprague–Dawley rats and hypoxia models of murine BV-2 microglia or PC12 cells under oxygen/glucose deprivation were used for *in vivo* and *in vitro* test, respectively. Lipid peroxidation and superoxide dismutase (SOD) activity from the ischemic brain were tested and reactive oxygen species (ROS), cytokine production, prostaglandin (PGE_2_) and related signaling pathways from hypoxic cells were examined by ELISA or Western blot assay, respectively.

**Results:**

BBD showed a protective effect when given 90 min after the focal cerebral ischemia. It also reduced lipid peroxidation and preserved SOD activity from the ischemic brain. The mechanism of BBD was further confirmed by attenuating ROS, cytokine production, and PGE_2_ release from hypoxic BV-2 or PC12 cells. BBD significantly reduced hypoxia-induced c-Jun N-terminal kinases (JNK) and modulated AKT-1 and caspase-3 (survival and apoptotic pathways) in BV-2 cells, and inhibited hypoxia-induced JNK and cyclooxygenase-2 activation in PC12 cells.

**Conclusions:**

The neuroprotective effect of BBD on ischemia/hypoxia models was involved with antioxidant and anti-inflammatory effects. The result would help the development of new CNS drug for protection of ischemia/hypoxia injury.

## Background

Stroke is the most common disease in the elderly population. Ischemic stroke is commonly caused by thrombosis that results in acute cerebrovascular disease and the lack of glucose and oxygen would damage the neuronal cells. In Taiwan, cerebrovascular disease is one of the leading causes of death in recent years (Department of Health, Taiwan). Brain ischemia/hypoxia is characterized by an increase reactive oxygen species (ROS) generation and cytokine-mediated inflammatory reactions [1]. Studies have shown that ischemia/reperfusion of brain can cause cell damage by increasing inflammation from oxidative stress [2,3]. Previously we reported that sesamin protected cerebral ischemia and neuronal cell injuries under stress [4,5]. However, sesamin might not penetrate the BBB easily because it has to be pretreated for its neuroprotective effect to ischemia/hypoxia-induced injuries [4-6]. A good neuroprotective agent should be able to pass the blood–brain barrier (BBB) to reach the brain target site [7,8].

**Figure 1:**
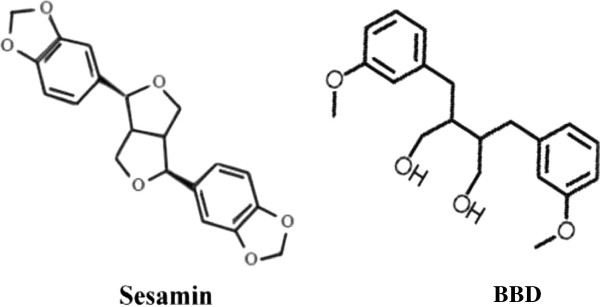
The chemical structure of sesamin and BBD.

Ischemia/hypoxia-induced ROS and cytokine can be scavenged by antioxidants [9]. Rat pheochromacytoma (PC12) cells and murine microglia BV-2 cells have been used as neuronal stress models [4,5,10]. Specifically, extracellular signal-regulated kinase (ERK), c-Jun N-terminal kinase (JNK) and p38 mitogen-activated protein kinase (MAPK) signaling pathways can be activated by ROS in PC12 cell and BV-2 cells [4,5]. Hypoxia-ischemia induces apoptosis in the brain is evident by release of cytochrome c and activation of caspase-3 [11]. Therefore in the present study, a compound, 3-bis (3-methoxybenzyl) butane-1,4-diol (BBD, Figure 1), with high membrane permeability was selected from a panel of newly synthesized sesamin derivatives to test its neuroprotective effect. The possible mechanism of BBD was investigated with ischemic brain and hypoxia models under oxygen and glucose deprivation (hypoxia) for ROS, cytokine, and PGE_2_ production. Hypoxia-induced MAPKs, apoptotic pathways, and COX-2 were also studied.

## Methods

### Reagents

Dimethylsulfoxide (DMSO), lucifer yellow, n-Dodecane, phosphate buffered saline (PBS), theophylline, and verapamil were obtained from Sigma-Aldrich Chemical (St. Louis, MO, USA). Porcine polar brain lipid (PBL) was purchased from Avanti Polar Lipids Inc. (Alabaster, AL, USA). 2′,7′-Dichlorodihydrofluorescein diacetate (H_2_DCF-DA) was obtained from Molecular Probe (Eugene, Oregon, USA). Fetal bovine serum (FBS) was obtained from Gibco Invitrogen (Grand Island, NY, USA). Dulbecco’s Modified Eagle’s medium (DMEM) were purchased from GIBCO (Grand Island, NY, USA). Anti-phospho-p38, ERK, JNK, and β-actin antibodies were purchased from Abcam (Cambridge, UK). Anti-Akt1 (pSer^473^) antibody was purchased from Calbiochem (Darmstadt, Germany). 3-bis (3-methoxybenzyl) butane-1,4-diol (BBD) was kindly provided from Joben Bio-Medical Co. (Kaohsiung, Taiwan).

### Membrane permeability assay

The blood–brain barrier limits drug access into the brain, due to tight junctions, membrane drug transporters, and unique lipid composition. Porcine whole-brain lipid is successfully used in passive permeability test for CNS drugs [7,8].

The parallel artificial membrane permeation assay (PAMPA) was carried out in a sandwich-like 96-well PAMPA plate formed by a top filter plate containing acceptor wells and a bottom plate containing donor wells. Each composite well was divided into two chambers: 300 μl of donor solution (test article or QC standards) at the bottom and 200 μl of acceptor buffer (PBS buffer containing 5% DMSO) at the top, separated by a polyvinylidene fluoride (PVDF, 0.45 μm pore size) membrane, coated with 4 μl of 20 mg/ml porcine polar brain lipid (PBL) in n-dodecane. Stock solutions of 2 mg/mL of a sesamin derivative (BBD), theophylline (low permeability standard), verapamil (high permeability standard), and lucifer yellow (used to assess lipid layer integrity in donor well) were prepared in DMSO and then diluted 20-fold in PBS buffer at pH 7.4 to yield final concentrations of 100 μg/ml of each of working stock solutions. The final DMSO concentration in working stock solution is 5%. Each compound was performed in triplicate. The test compound diffused from the donor well through the lipid membrane and into the acceptor well. The sandwich plate was placed with the plate lid and incubated at room temperature for approximately 18 h. After reaching diffusion equilibration, the PAMPA sandwich plate was disassembled and the concentration of drug in the acceptor, the donor, and the reference wells was determined using a TECAN microplate reader (Durham, NC, USA). Effective permeability (P_e_) of the compounds was calculated using the Microsoft Office Excel 2010. The range for P_e_ of a BBB + compound (high BBB permeation predicted): P_e_ (10^-6^ cm s^-1^) > 4.0; BBB- compound (low BBB permeation predicted): P_e_ (10^-6^ cm s^-1^) < 2.0; and BBB+/- compound (BBB permeation uncertain): P_e_ (10^-6^ cm s^-1^) from 4.0 to 2.0 [7].

### Animal study

Twenty-four male Sprague–Dawley (SD) rats (200~250 gm) were purchased from National Animal Center, Taipei, Taiwan and randomly divided into the control (normal saline), and BBD groups. The experiment was approved by Institutional Animal Care and Use Committee, Taichung Veterans General Hospital (IACUC LA-97490). Rats were injected intraperitoneally (i.p.) with BBD (10 mg/kg) 90 min after MACO experiment. Each SD rat was anesthetized with chlorohydrate (400 mg/kg) i.p. and its body temperature was maintained at 37°C with a heating pad (CMA/150). A midline neck incision was made and the right carotid artery was exposed and separated from the vago-sympathetic trunk. The right carotid artery was loosely encircled with a 4-O suture for later occlusion. The SD rat’s head was placed in a stereotaxic frame (David Kopf, CA, USA) with the nose bar positioned 4.0 mm below the horizontal line. Following a midline incision, the skull was partially removed to expose the right middle cerebral artery. The middle cerebral artery was loosely encircled with an 8-O suture for later occlusion. A focal cerebral ischemia was induced by occlusion of the right common carotid artery and the right cerebral artery (MCAO) for 60 min, followed by reperfusion. A laser probe (0.8 mm in diameter) of a Laser Doppler Blood Flow monitor (MBF 3D, Moor Instruments, Axminster, UK) was positioned onto the cortex with its tip close to the middle cerebral artery. Cerebral blood flow dropped to less than 5% of basal after the occlusion of the MCAO. Cerebral blood flow reached its minimal levels within 5 min after the start of the occlusion and was confirmed to remain at this level throughout the monitoring period to ensure the validity of the stroke model. Twenty-four hours after cerebral ischemia, each SD rat was anesthetized and perfused transcardially with isotonic heparinized saline and 2,3,5-triphenyltetrazolium chloride (TTC). The brain was then removed and sliced into five 2-mm-thick coronal sections for TTC staining.

### Cell culture

Murine BV-2 microglial cell line was maintained in DMEM supplemented with 10% (v/v) FBS, 100 U/ml penicillin and 100 μg/ml streptomycin (P/S) in a humidified incubator under 5% CO_2_ at 37°C. Rat pheochromacytoma PC12 cell line was maintained in DMEM supplemented with 10% FBS, 5% horse serum, P/S at 37°C under 5% CO_2_. Confluent cultures were passaged by trypsinization. In all experiments, the cells were treated with BBD immediately before hypoxia. BBD was dissolved in DMSO. The final concentration of DMSO added to cells never exceeded 0.1% (v/v).

### Hypoxia

On the day of experiment, culture media were replaced with glucose-free DMEM, then, gassed with 85% N_2,_ 10% H_2_, and 5% CO_2_ for various time periods in the absence or presence of various doses of BBD.

### MTT assay

Cell viability was measured using blue formazan that was metabolized from colorless 3-(4,5-dimethyl-thiazol-2-yl)-2,5-diphenyl tetrazolium bromide (MTT) by mitochondrial dehydrogenases, which are active only in live cells. PC12 or BV2 cells were preincubated in 24-well plates at a density of 5 × 10^5^ cells per well for 24 h. Cells incubated with various concentrations of BBD were under hypoxia for 30 min, and incubated in 0.5 mg/ml MTT at 37°C. One hour later, 200 μl of solubilization solution were added to each well and absorption values read at 540 nm on microtiter plate reader (spectraMAX 340, Molecular Devices, Sunnyvale, CA, USA). Data were expressed as the mean percent of viable cells vs. control.

### LDH assay

Cytotoxicity was determined by measuring the release of LDH. PC12 or BV-2 cells treated with various concentrations of BBD were stressed with hypoxia for one hour and the supernatant was then assayed for LDH activity. An absorbance was read at 490/630 nm using a spectraMAX 340 microtiter plate reader. Data were expressed as the mean percent of viable cells vs. the control.

### Generation of reactive oxygen species

Intracellular accumulation of ROS was determined using H_2_DCF-DA, which is a nonfluorescent compound that accumulates in cells following deacetylation. H_2_DCF then reacts with ROS to form fluorescent dichlorofluorescein (DCF). PC12 cells were plated in 96-well plates and grown for 24 h before addition of DMEM plus 10 μM H_2_DCF-DA, incubated for 60 min at 37°C, and treated with various concentrations of BBD for hypoxia 30 min. Cells were then washed twice at room temperature with Hank’s balanced salt solution (HBSS without phenol red). Cellular fluorescence was monitored on a Fluoroskan Ascent fluorometer (Labsystems Oy, Helsinki, Finland) using an excitation wavelength of 485 nm and emission wavelength of 538 nm.

### Measurement of cytokine assay

Cytokines (IL-1β, IL-6) and PGE_2_ were measured using ELISA kits (R&D, Minneapolis, MN, USA). The absorbance at 450 nm was determined using a microplate reader (spectraMAX 340).

### Western blot

Samples containing 25 μg of protein were separated on 12.5% (w/v) sodium dodecyl sulfate-polyacrylamide gels, and transferred to immobilon polyvinylidenedifluoride membranes (Millipore, Bedford, USA). The membranes were incubated for 2 h with 5% (w/v) dry skim milk in TBST buffer to block non-specific binding, then ERK, p38 JNK, AKT-1, COX-2, caspase-3, β-actin proteins for neuron cells were detected by a chemiluminescence detection system according to the manufacturer’s instructions (ECL, Amersham, Berkshire, UK).

### Superoxide dismutase (SOD) assay

Superoxide dismutase (SOD) activity was determined by a Superoxide Dismutase assay kit (Cayman, Ann Arbor, MI, USA). This method was based on the formation of red formazan from the reaction of 2- (4-iodophenyl) -3- (4-nitrophenol) -5-phenyltetrazolium chloride and superoxide radical and assayed in a spectrophotometer at 505 nm. The inhibition of the produced chromogen was proportional to the activity of the SOD present in the sample. A 50% inhibition was defined as one unit of SOD, and the specific activity was expressed as units per milligram protein.

### Lipid peroxidation

Lipid peroxidation is quantified by measuring malondialdehyde (MDA) of PC12 cells and brain tissue of SD rats by lipid peroxidation (LPO) assay kit (Cayman). This kit works on the principle of condensation of one molecule of either MDA or 4-hydroxyalkenals with two molecules of N-methyl-2-phenylindole to yield a stable chromophore. MDA levels were assayed by measuring the amount expressed in 5 × 10^5^ cells of PC12 and SD brain tissue, and the absorbance at 500 nm was determined using a microplate reader (spectraMAX 340).

### Statistical analysis

Data were expressed as the mean ± SEM. In animal study, TTC data were analyzed by analysis of variance (ANOVA) with Student’s *t*-tests. A *P* value less than 0.05 was considered to be statistically significant. For *In vitro* study with single variable comparisons, Student’s *t*-test was used. For multiple variable comparisons, data were analyzed by one-way ANOVA followed by Scheffe’s test.

## Results

### *In vivo* effect of BBD on the cerebral ischemia

**Figure 2:**
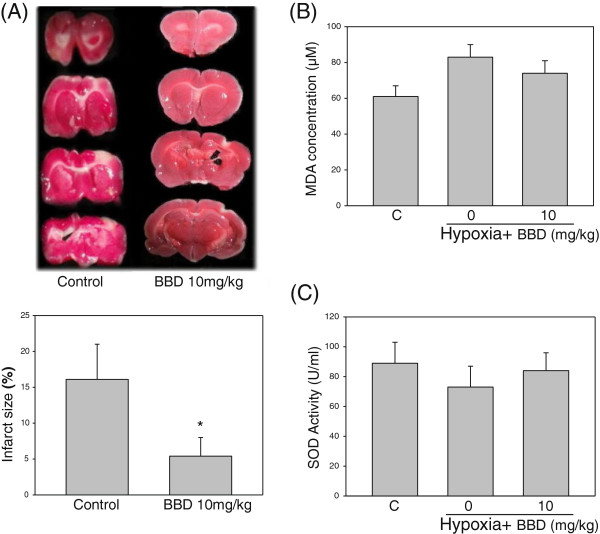
***In vivo *effect of BBD on the cerebral ischemia.** SD rats treated with BBD i.p. 90 min after MACO had reduced infarct sizes of brains **(A)**. Data are expressed as mean ± SEM. ^*^: *P* < 0.05 as compared to the non-treated ischemic control. (n = 8). SOD activity and lipid peroxidation were determined by commercial assay kits (Cayman, Ann Arbor, MI, USA). Malondialdehyde (MDA) of SD rats was increased by ischemic stress but reduced by BBD **(B)** and SOD activity was restored by BBD **(C)**. Data are expressed as mean ± SEM. ^*^: *P* < 0.05 as compared to the hypoxia control. (n = 4).

BBD had a high membrane permeability by PAMPA assay and was regard as a BBB permeable agent (Table 1). SD rats treated i.p. with BBD (10 mg/kg) 90 min after MCAO-induced ischemia reduced 66% of the infarct size as compared to the cerebral ischemia group (from 16.1 to 4.9%, *P* < 0.05; Figure 2A). MDA level of the BBD group was decreased 6% (from 83 μM to 78 μM) as compared to the ischemia group (Figure 2B). BBD treatment increased a 24% SOD activity (from 68 μ/mL to 84 μ/mL) as compared to the ischemia group (Figure 2C).

**Table 1:** The PAMPA-BBB permeation of BBD

**Compound**	**Pe (10**^**-6**^ **cm/s)**	**Known Pe (10**^**-6**^ **cm/s)**	**Classification**
Theophylline	0.08 ± 0.01	0.12 ~ 0.18	BBB-
Verapamil	23.82 ± 1.82	16 ~ 23	BBB+
Testosterone	34.08 ± 4.78	17	BBB+
BBD	20.87 ± 3.05	-	BBB+
Sesamin	0.00 ± 0.01	-	BBB-

### Protection of hypoxic damage

**Figure 3:**
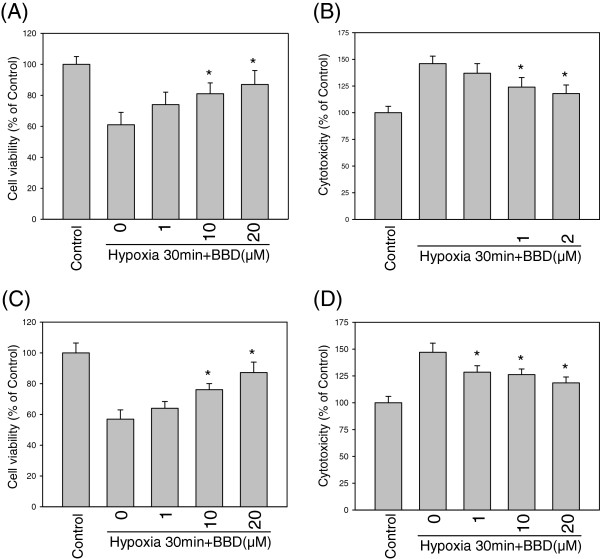
**Effect of BBD on cell viability and cytotoxicity of PC12 and BV-2 cells under hypoxia.** The cells were treated with hypoxia alone or in the presence of various concentrations (1, 10, 20 μM) of BBD for 30 min. Cell viability of PC12 **(A)** and BV-2 cells **(C)** was increased and the LDH release, reduced **(B, D)** from hypoxia dose-dependently by BBD. Data are expressed as mean ± SEM of three independent experiments in triplicate. ^*^: *P* < 0.05 as compared to the hypoxia control.

In order to investigate the protective mechanism of BBD, an *in vitro* hypoxia model was studied with neuronal cell-lines. Hypoxia can induce free radicals and damage neuronal cells, therefore the cell viability and LDH released from PC12 and BV-2 cells were measured using MTT and LDH ELISA assays. As shown in Figure 3A, the cell viability of PC12 cells under hypoxia for 30 min was preserved by the presence of BBD (1 to 20 μM). Hypoxia-induced LDH released was also decreased by BBD treatment (*P* < 0.05, Figure 3B). Similarly, BV-2 cells were protected by BBD under hypoxia (*P* < 0.05, Figure 3C,D).

### ROS scavenging effect of BBD

**Figure 4:**
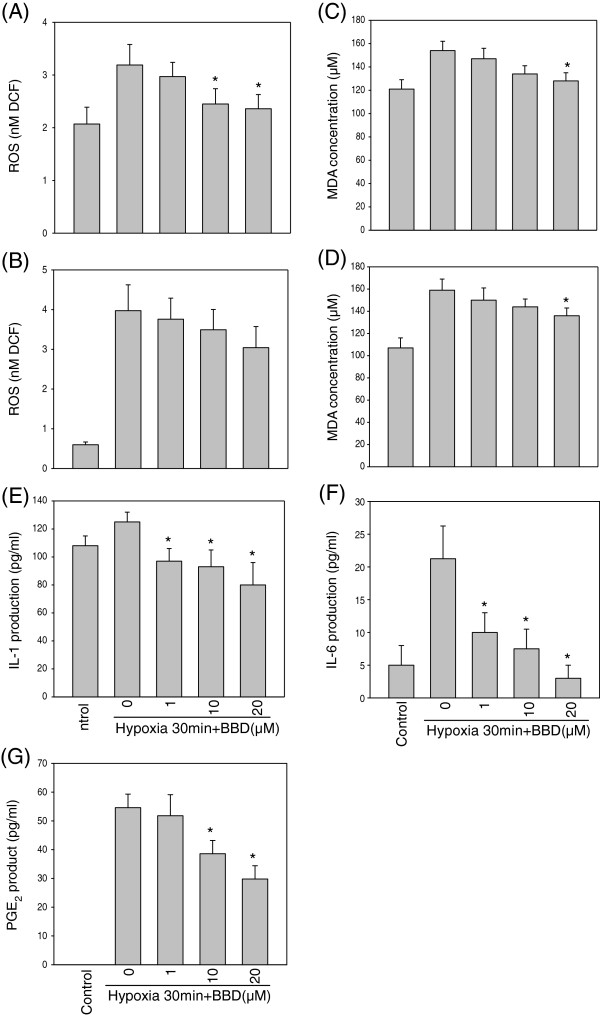
**Effects of BBD on hypoxia-induced ROS generation, IL-1, IL-6 and PGE**_**2 **_**productions.** PC12 and BV-2 cells were treated with 1, 10, 20 μM of BBD for 30 min. BBD reduced the generation of ROS under hypoxia in PC12 **(A)** and BV-2 **(B)** cells. MDA of PC12 **(C)** and BV-2 **(D)** cells were induced by 30-min hypoxia and reduced by BBD. BV-2 cells were treated with 1, 10, 20 μM of BBD under hypoxia 30 min. BBD concentration-dependently reduced hypoxia-induced IL-1 **(E)**, IL-6 **(F)** and PGE_2_**(G)** production from BV-2 cells. Data are expressed as mean ± SEM of 3 independent experiments in triplicate. ^*^: *P* < 0.05 as compared to the hypoxia control, n = 3.

Under hypoxia, ROS (as DCF signal) was increased nearly half (in PC12 cells) to four-fold (in BV-2 cells) as compared with their control cells. BBD protected cells against hypoxia-induced cell toxicity by decreasing the ROS accumulation in both cells (Figure 4A,B). The increase in MDA level was suppressed by BBD in hypoxia-exposed PC12 or BV-2 cells as compared with the control cells (Figure 4C,D, *P* < 0.05).

### BBD inhibited IL-1, IL-6 and PGE_2_

BBD dose-dependently decreased the production of the inflammatory cytokine, IL-1 and IL-6 from BV-2 cells under hypoxia (*P* < 0.05, Figure 4E,F). We further evaluated the effect of BBD on hypoxia-induced PGE_2_ production. BV-2 cells were incubated with 1, 10, 20 μM of BBD then subjected to hypoxia for 30 min. The results showed that BBD (10 and 20 μM) decreased PGE_2_ release from BV-2 cells significantly (*P* < 0.05, Figure 4G).

### BBD inhibited hypoxia-induced JNK MAPK, COX-2 and caspase-3 activation

**Figure 5:**
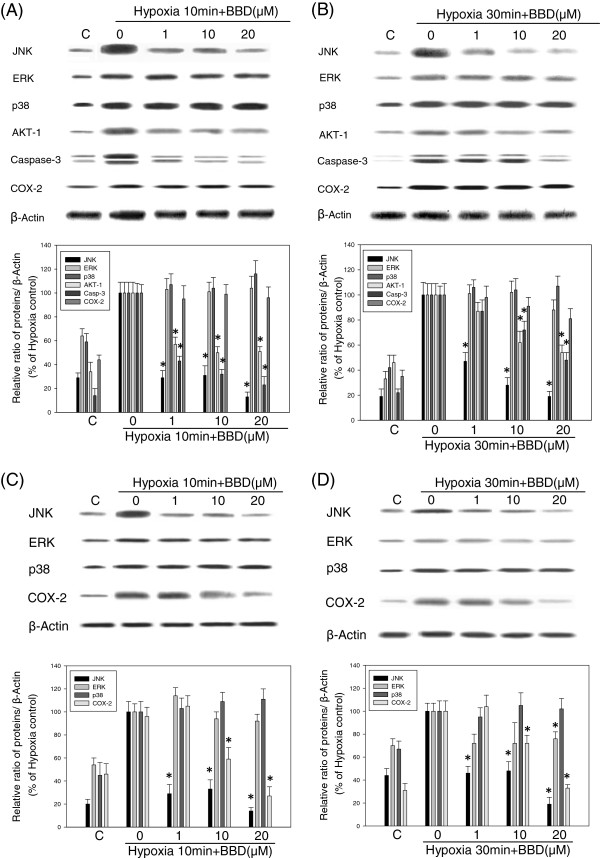
**BBD inhibited hypoxia-induced phospho-JNK MAP kinase, AKT-1, COX-2 and Caspase-3 under hypoxia.** The effect of BBD (1, 10, 20 μM) on hypoxia-activated cell signaling pathways was determined by Western blotting under hypoxia stress in BV-2 cells for 10 min **(A)** and for 30 min **(B)**. Similarly, BBD effectively reduced the activation of JNK MAP kinase and COX-2 **(C, D)** in PC12 cells. C: normal control. Data are expressed as mean ± SEM of three independent experiments. ^*^: *P*<0.05 as compared to hypoxia control.

The effects of BBD (1, 10, 20 μM) on hypoxia-induced signaling pathways were further examined by Western blot assay (Figure 5). BBD (20 μM) reduced expression of the following proteins: JNK (88 ± 6%), ERK (10 ± 7%), p38 (-8 ± 10%) MAPKs, AKT-1 (45 ± 6%), Caspase-3 (50 ± 7%), and COX-2 (13 ± 7%), respectively to the 10 min hypoxia-induced BV-2 cells (*P < 0.05; Figure 5A). This result is better than that of the 30 min hypoxia-induced BV-2 cells (*P < 0.05; Figure 5B). Similarly, BBD (20 μM) also suppressed hypoxia induced expression of the signaling proteins in PC12 cells: JNK (86 ± 5%), ERK (9 ± 7%), p38 (-10 ± 9%) MAPKs, and COX-2 (75 ± 11%), respectively (*P < 0.05; Figure 5C). This was better than that of the 30 min hypoxia-induced PC12 cells (*P < 0.05; Figure 5D).

## Discussion

The present study showed that BBD could pass the BBB by PAMPA assay and significantly protected animals from the focal cerebral ischemia. Furthermore, BBD was able to suppress MDA and preserve SOD activity in the ischemic rat brain. BBD at the concentrations of 10 to 20 μM, decreased hypoxia- induced cell viability, ROS generation and MDA levels in BV-2 and PC12 cells.

Excessive ROS production in the brain is believed to contribute to neurodegenerative processes [12,13]. Various dietary-derived antioxidants that inhibit the hypoxia-induced inflammation response may have neuroprotective potential [14,15]. Since sesamin and its related structure were reported to have protective effect on the hypoxia-induced inflammatory and oxidative stress [5,16], BBD, a sesamin derivative would have a similar effect. Effect of BBD on hypoxia-induced MDA stress might be through the activation of antioxidant signaling pathway such as Nrf2/ARE [17].

We found that 10 to 30 min hypoxia could significantly induce the activation of JNKs, AKT-1, and caspase-3 expression in BV-2 cells and JNK, ERK, COX-2 expression in. PC12 cells. Inhibition of JNK MAPK, COX-2 and caspase-3 can be expected to be beneficial in injuries involving microglia activation and inflammation. Specific inhibitors of JNK MAPK have been proven to reduce inflammation, slow down microglia activation and provide neuroprotective effects [4,18,19]. Studies have shown that antioxidant compounds inhibit JNK MAPK activation in microglia represent potential anti-inflammatory effects and protect neurons damage [5,20,21]. In addition, antioxidant compounds inhibit JNK MAPK activation in neuron and cardiomyocyte cells represent potential protective effects from hypoxic damage [5,22-24]. Sesamin can regulate microglial activities by inhibition of the intracerebral hemorrhage-induced p44/42 MAPK pathway and protect neuronal cells by inhibition of hypoxia-induced ERK, JNK, p38 MAPK [5,25]. BBD, a sesamin derivative also suppressed hypoxia-induced JNK MAPK expression in both cells significantly. Studies have shown that hypoxia induces MAPK activation and apoptosis factor Caspase-3 *in vitro* and *in vivo*[4,11]. Therefore, we evaluated the effect of BBD on hypoxia-induced signaling pathways including MAP kinases (JNK, ERK, p38), AKT-1, Caspase-3 and COX-2. Western blot analysis revealed that BBD (10 and 20 μM) significantly reduced JNK MAPK, AKT-1 and Caspase-3 expression in BV-2 cells as compared to hypoxia controls (Figure 5A,B). Similarly, BBD significantly reduced JNK MAPK and COX-2 expression in PC12 cells with both 10 and 30 min hypoxia as compared to hypoxia controls (Figure 5C,D). The results suggested that BBD restored the cell viability under hypoxic stress (Figure 3) through various pathways in each cells. This also agrees with a recent study that agent protects neuronal cells from H_2_O_2_-induced cell death, DNA fragmentation, and activation of caspase-3 and MAP kinase can ameliorate ischemic brain injury [26].

Induction of antioxidant enzymes has been considered as a promising strategy to combat with oxidative stress-related diseases. Previous studies shown that neuroprotective effects of antioxidants are due to increasing the level of antioxidant enzymes (e.g., SOD, catalase, G6PD), lowering of ROS, and preventing calcium release [5]. SOD is an important enzyme for eliminating free radicals and protect brain tissues from the ischemic injury [27]. Recently a study shows that sesamin and metabolites induce phase II antioxidant enzymes such as heme oxygenase-1 (HO-1) by activation of Nrf2/ARE (antioxidant response element) signaling and suggesting their potential to reduce oxidative stress and ameliorate oxidative stress-related neurodegenerative diseases [17]. Since BBD was able to suppress MDA and preserve SOD activity in the ischemic rat brain and inhibited 40-50% of hypoxia-induced ROS, IL-1, and IL-6 production, it might also activate this antioxidant signaling pathway, and awaits future study.

ROS could induce cell damage by activating MAPK, and the nuclear transcription factor c-Jun [28]. The downstream of ROS signaling pathway could be associated with microglia activation. Since ROS are cytotoxic mediators in microglia [28]. BBD may also down-regulate hypoxia-induced inflammatory factor production via the inhibition of ROS generation which would reduce the activation of IL-1 and IL-6 cytokines in BV-2 cells. The abilities of BBD to inhibit the hypoxia-induced COX-2 protein might be due to decreased attenuation of ROS signal, and reduced JNK MAPK in PC12 cells. Caspase-3 is an important apoptosis factor for neuronal cells [26]. Application of BBD (100 μM) alone was not toxic to neurons (data not shown) and BBD at the lower concentration (20 μM) inhibited the inflammation response in BV-2 and PC12 cells under hypoxia. BBD significantly reduced infarct volume (about 66%) of ischemic brain in SD rats as compared to the control group. Although the precise mechanism of BBD neuroprotection is not clear, the present *in vitro* and *in vivo* results suggest that its protection might be involved with the inhibition of release of ROS and inflammation during cerebral ischemia.

## Conclusion

In conclusion, the present study shows that BBD with a high membrane permeability protected the brain after the focal cerebral ischemia. It also reduced lipid peroxidation and preserved superoxide dismutase activity from the ischemic brain. The protective mechanisms of BBD might be involved with the inhibition of JNK MAPK, COX-2, and caspase-3 signal pathway. These results extend our knowledge of BBD to its therapeutic potential.

## Abbreviations

MTT: 3-(4,5-dimethyl-thiazol-2-yl)-2,5-diphenyl tetrazolium bromide; BBD: 3-bis (3-methoxybenzyl) butane-1,4-diol; JNK: c-Jun N-terminal kinase; COX: Cyclo-oxygenase; ERK: Extracellular signal-regulated kinase; p38 MAPK: p38 mitogen-activated protein kinase; PGE2: Prostaglandin E2; hypoxia: Oxygen/glucose deprivation; ROS: Reactive oxygen species; SOD: Superoxide dismutase.

## Competing interests

The authors declare that they have no competing interests.

## Authors’ contributions

C-WH participated in the design, infarction studies, data processing and manuscript preparation. Y-LC participated in animal procedures, SOD and MDA assay, and Western blot and *in vitro* assay. S-H Chuang participated in the PAMPA-BBB permeation assay. J-S Wang participated in the discussion. K-CJ prepared the final manuscript. All authors read, discussed and approved the final manuscript.
